# Integrated System Design: Promoting the Capacity of Sociotechnical Systems for Adaptation through Extensions of Cognitive Work Analysis

**DOI:** 10.3389/fpsyg.2016.00962

**Published:** 2016-06-28

**Authors:** Neelam Naikar, Ben Elix

**Affiliations:** Defence Science and Technology GroupMelbourne, VIC, Australia

**Keywords:** system design, adaptation, self-organization, sociotechnical system, cognitive work analysis

## Abstract

This paper proposes an approach for integrated system design, which has the intent of facilitating high levels of effectiveness in sociotechnical systems by promoting their capacity for adaptation. Building on earlier ideas and empirical observations, this approach recognizes that to create adaptive systems it is necessary to integrate the design of all of the system elements, including the interfaces, teams, training, and automation, such that workers are supported in adapting their behavior as well as their structure, or organization, in a coherent manner. Current approaches for work analysis and design are limited in regard to this fundamental objective, especially in cases when workers are confronted with unforeseen events. A suitable starting point is offered by cognitive work analysis (CWA), but while this framework can support actors in adapting their behavior, it does not necessarily accommodate adaptations in their structure. Moreover, associated design approaches generally focus on individual system elements, and those that consider multiple elements appear limited in their ability to facilitate integration, especially in the manner intended here. The proposed approach puts forward the set of possibilities for work organization in a system as the central mechanism for binding the design of its various elements, so that actors can adapt their structure as well as their behavior—in a unified fashion—to handle both familiar and novel conditions. Accordingly, this paper demonstrates how the set of possibilities for work organization in a system may be demarcated independently of the situation, through extensions of CWA, and how it may be utilized in design. This lynchpin, conceptualized in the form of a diagram of work organization possibilities (WOP), is important for preserving a system's inherent capacity for adaptation. Future research should focus on validating these concepts and establishing the feasibility of implementing them in industrial contexts.

## Introduction

This paper is concerned with the design of sociotechnical systems, particularly those that are complex in nature (Vicente, 1999), such as hospitals, nuclear power plants, petrochemical refineries, military ships and aircraft, emergency management centers, and financial corporations. Designing such systems, which perform vital functions for people and society, poses considerable challenges, not least because the stakes are high—patients' lives must be saved, enemy attacks must be deterred, and natural disasters must be contained. High levels of productivity must be balanced with high levels of safety and reliability, often with shortfalls in resources, whether this is in equipment or in personnel. It is not uncommon, therefore, for these systems to operate at the edges of their effectiveness, with a fine line between successful performance and disastrous consequences. Moreover, in cases of failure, poor design has often been established as a significant contributor, with examples of such accidents including the delivery of fatal radiotherapy or chemotherapy overdoses to patients (Leveson and Turner, 1993; Institute for Safe Medication Practices, 2007), crashes of commercial airliners resulting in the deaths of hundreds of passengers and crew (Bureau of Enquiry and Analysis for civil aviation safety, 2002, 2012), military fratricide (32nd Army Air Missile Defense Command, 2003), and oil and petrochemical explosions with widespread consequences for people, infrastructure, and the natural environment (Mannan et al., 2007). Evidently, then, the question of which design philosophy and methods should underpin how these systems are conceived or formed should not be made arbitrarily.

The approach for integrated system design presented in this paper subscribes to the view that the fundamental objective in designing sociotechnical systems should be that of promoting adaptation, so that workers can deal with both routine and novel events effectively. Thus the paper begins by summarizing the empirical observations in support of this basic argument, originally formulated by Rasmussen and his colleagues (e.g., Rasmussen, 1986; Rasmussen et al., 1994). Subsequently, a case is made that designs must support actors in adapting not only their behavior but also their structure, or organization. While the importance of structural adaptation has not been unappreciated before, existing approaches for work analysis and design are limited in their capacity to support this form of adaptation. The argument is then developed, following Vicente (2002), that in designing for adaptation it is insufficient to focus on individual system elements, such as the interfaces, teams, training, or automation. Rather, the design of multiple elements must be integrated, or coordinated, such that workers are supported in adapting their structure and behavior in a coherent fashion. This paper therefore examines the capacity of current frameworks for work analysis and design to meet this objective, focusing on cognitive work analysis (CWA). Following that, the integrated system design approach is presented, which extends CWA with the intent of meeting this critical goal.

## Designing for adaptation

### Importance of adaptation in the workplace

A strong case has already been made that the fundamental objective in designing complex sociotechnical systems should be that of promoting successful adaptation (Rasmussen, 1986; Rasmussen et al., 1994; Vicente, 1999). This thesis, which manifests widely in one form or another (e.g., Dekker, 2003; Hollnagel et al., 2006, 2011; Hoffman and Woods, 2011; Eason, 2014; Rankin et al., 2014), is supported by a number of empirical observations.

First, complex sociotechnical systems are by and large open systems, characterized by changing or dynamic conditions (Ashby, 1956; Emery and Trist, 1965; Perrow, 1984; Gerson and Star, 1986; Rasmussen, 1986; Rasmussen et al., 1994; Vicente, 1999). This instability may result from regular perturbations, either within the system (e.g., technical malfunctions, staffing shortages) or in the external environment (e.g., economic fluctuations, changing weather patterns). Moreover, these systems may have to contend with novel circumstances, or events that cannot be fully predicted a priori, such as a new kind of military threat (Reich et al., 2010; Herzog, 2011), an unexpected reaction of a patient to an anesthetic during surgery (Hoppe and Popham, 2007), or an unforeseen chain of supplier collapses in the wake of a natural disaster (Park et al., 2013). These systems, therefore, must be capable of continuously and reliably dealing with significant variability in their work environments.

Studies of complex sociotechnical systems have also demonstrated that the greatest threats to these systems' effectiveness are posed by unanticipated events (e.g., Rasmussen, 1968a,b, 1969; Perrow, 1984; Reason, 1990; Leveson, 1995; Vicente, 1999). As these situations cannot be predicted, analysts or designers cannot provide workers with “ready-made” solutions for handling these events. Moreover, as these situations are unfamiliar to workers, they cannot simply retrieve a suitable solution from their portfolios of prior experiences. Instead, workers must respond flexibly and creatively to deal with these situations successfully (e.g., Rochlin et al., 1987; Bigley and Roberts, 2001; Bogdanovic et al., 2015) and thus finish the design (Rasmussen and Goodstein, 1987).

Aside from dealing with unexpected events, adaptations are necessary regularly, or even routinely, in everyday situations (Simon, 1969; Gerson and Star, 1986; Rasmussen, 1986; Suchman, 1987; Weick, 1993; Rasmussen et al., 1994; Vicente, 1999). Even small changes in context may require adaptation (Vicente, 1999), and it is not possible to formulate an algorithm, plan, or procedure for every single complication (Hoffman and Woods, 2011), even if it were safe to do so (Dekker, 2003). Thus everyday work requires ongoing local adjustments or improvisations to accommodate the inevitable flux that arises in the system (Bigley and Roberts, 2001; Rankin et al., 2014; Bogdanovic et al., 2015; Militello et al., 2015).

Another significant observation is that adaptations are important not just for safety but also for organizational productivity and workers' health (Vicente, 1999). In computerized workplaces, where routine tasks are typically automated, system success can hinge on the capacity of workers to conjure up innovative solutions to emerging problems for which algorithms have not been, or cannot be, written. Furthermore, it has long been recognized that workers with greater decision latitude tend to have better health, as indicated by such factors as longevity and the absence of stress or disease (Karasek and Theorell, 1990; Vicente, 1999; Eason, 2014). Such workers have the autonomy to decide how to manage their work demands, including the ability to improvise or adapt in doing their jobs, and to follow their individual preferences when it is appropriate to do so.

Finally, while the importance of adaptation in the workplace is clear, it is also evident that ongoing adaptation to changing situations and unforeseen circumstances can be demanding (Rasmussen, 1986; Rasmussen et al., 1994; Vicente, 1999; Dekker, 2003; Hoffman and Woods, 2011; Bogdanovic et al., 2015). The context or conditions under which adaptation is required, as it is experienced by workers, is usually exacting, involving multiple, conflicting goals, significant time pressure, many unexpected turns of events, and considerable stress stemming from the awareness of the potentially disastrous consequences of failure. Furthermore, adaptation can be an intellectually or cognitively challenging exercise, involving very complex reasoning under demanding conditions (Rasmussen et al., 1994; Dörner, 1996; Vicente, 1999). Typically, workers must make rapid decisions about whether, when, and how to adapt in light of their judgments of the local conditions, awareness of the broader organizational goals and constraints, and assessments of the risks and opportunities this context presents (Dekker, 2003).

Workers, therefore, should not have to—or be expected to—adapt in an *ad hoc* manner, using technology or workplace designs that do not support or, worse still, deliberately inhibit improvisation, as is so often the case (Vicente, 1999; Eason, 2014). Aside from placing, quite unnecessarily and unfairly, an increased burden on workers who are already working under very demanding conditions, this situation could lead or contribute to unsafe or unproductive outcomes. Instead, workers should be provided with systematic support through the system design, including the design of technology, training, and procedures, to help them in adapting seamlessly and successfully to the unexpected and changing demands of their jobs (Rasmussen, 1986; Rasmussen et al., 1994; Vicente, 1999; Dekker, 2003; Eason, 2014; Rankin et al., 2014; Militello et al., 2015).

### Behavioral and structural adaptation

If we are to design systems that facilitate successful adaptation, a key question that arises is what manner of adaptations are needed in the workplace, and thus should be deliberately supported through design. The following studies demonstrate the importance of both behavioral and structural adaptation to system effectiveness. Greater emphasis is placed on illustrating the nature of structural adaptation in the workplace, since existing analysis and design approaches are limited in supporting this form of adaptation, as discussed in more detail later in this paper.

Empirical studies of workers in complex sociotechnical systems reveal that one form of adaptation that occurs entails actors adapting their behavior, or effectively adjusting their tasks, plans, goals, actions, or priorities in step with the unfolding situation. Bigley and Roberts (2001) provide a detailed account of the improvisations they observed during a field study of a large fire department employing the incident command system, a widespread approach for emergency management in the United States of America. They categorized the improvisations as involving tools, rules, and routines. When a truck arrives at the scene of an emergency, for instance, personnel may have no choice but to improvise with the tools available on the truck, employing them in unusual ways to handle the situation. In other cases, the adaptations may include departures from rules, directly breaching standard operating procedures. As an example, one procedure prohibits firefighting teams from approaching a fire from opposite positions, as one group can push the fire into another. However, a firefighter discussed a situation in which “opposing hose streams” was in fact used as the primary tactic. Lastly, the execution of standard routines, such as those for “hose laying” or “ladder throwing,” may also be adjusted to accommodate local contingencies. According to Bigley and Roberts, such improvisations are regarded as legitimate within the organization, provided they are consistent with organizational goals and are unlikely to harm personnel or other people.

Observations of behavioral adaptation in the workplace have also been documented in a number of other contexts. Goteman and Dekker (2001), for example, discuss how commercial pilots shed tasks when confronted with demanding circumstances, postponing some jobs until the situation becomes more manageable. Similarly, Militello et al. (2015) observed that military pararescue teams are constantly juggling priorities for evacuating injured personnel from hostile areas, depending on what transpires at the scene in relation to such factors as the urgency of patients' medical conditions, the actions of adversaries, and the available resources. Finally, within a health care context, Bogdanovic et al. (2015) discuss how surgeons may interrupt a surgical procedure on discovering unanticipated patient states, such as the presence of inflammation, in order to discuss the next steps with the medical team.

Further to such adaptations in workers' activities, empirical studies provide considerable evidence for structural adaptation, whereby multiple actors are involved in adjusting their structure or organization in line with the emerging situation. As a result, the particular actors involved and their roles and relationships may be constantly changing. A potent example is provided by Rochlin et al. (1987), who conducted a field study of how navy personnel on aircraft carriers coordinate their work activities. Rochlin et al. found that the formal organization of this system—that which is documented on paper—is rigid, hierarchical, and centralized, being characterized by clearly defined chains of command and means to enforce authority. Typically, this organizational structure governs operations on the ship.

During complex operations, however, Rochlin et al. (1987) found that a very different type of organizational structure is adopted. This organizational structure may be described as informal, given that it is not officially documented. The informal organization is flat and distributed rather than hierarchical and centralized. For instance, based on their access to information, lower-ranked personnel have the autonomy to make critical decisions without the approval of officials with higher rankings, especially when faced with significant time constraints. The informal organization is also flexible in that there is no pre-specified plan for when it will be adopted. Moreover, the specific organizational structure that is adopted on any one occasion is emergent, such that there is no simple or fixed mapping between people and roles and therefore no single informal organization. Instead, the work organization on the ship adapts to changes in circumstances. According to Rochlin et al. this adaptability contributes greatly to balancing the need for safety with the push for productivity.

Bigley and Roberts's (2001) observations of a fire department employing the incident command system for emergency management echo many of Rochlin et al.'s (1987) findings. At one level, this system is highly formalized with an extensive set of policies, procedures, and instructions. Jobs are specialized and have very particular training requirements. In addition, positions within the system are arranged hierarchically and reflect formal authority relationships. Objectives and plans are established near the top of the hierarchy and serve as a basis for guiding decisions and behaviors at lower levels. Nevertheless, as Bigley and Roberts discovered, the fire department consistently employs a number of mechanisms for rapidly converting this rigid organizational structure into highly flexible arrangements suitable for dealing with the specific emergencies encountered. Bigley and Roberts describe these mechanisms as involving structure elaborating, role switching, authority migration, and system resetting.

Structure elaborating describes the process of organization construction at the scene of an incident, with the first captain arriving becoming the incident commander, at least temporarily. After assessing the situation and developing an initial plan, the incident commander begins to build an organization by assigning roles and tasks to incoming resources, a process which may continue until the emergency shows signs of subsiding. Pre-existing roles or positions within the incident command system are filled with people only to the extent required, perhaps with more positions becoming filled as the situation unfolds. Furthermore, some functions may not be assigned to specialized positions until it is necessary to do so, with personnel already established in particular positions being responsible for multiple functions in the meantime.

Role switching sums up the observation that positions continue to be activated and relationships established in line with the emerging situation. In addition, positions are deactivated when the appropriate role structure for an emergency changes, and personnel are either shifted into different positions or discharged. Authority migration recognizes that although formal authority relationships remain fixed, informal decision-making authority can migrate rapidly to personnel possessing the most relevant expertise. Thus senior personnel may defer to lower-level experts who are more technically qualified given the specific characteristics of the emergency, temporarily shifting authority to them. Lastly, system resetting involves disengaging or regrouping. When the current approach appears to be having no effect or is found to be unsuitable because of unexpected occurrences, the team is withdrawn from the situation and reconfigured or redirected. As Bigley and Roberts observe, “Within the most reliable systems, objectives and corresponding structural elements and relationships are adjusted swiftly in accordance with changing environmental contingencies” (p. 1287).

Finally, Bogdanovic et al. (2015) provide a detailed account of how the task distribution among actors in surgical teams alters as a function of specific occurrences during surgery. According to Bogdanovic et al. only the general task distribution is established prior to the surgical procedure. While the delegation of some tasks are determined by team members' professions, such as whether one is an anesthetist, nurse, or surgeon, tasks that can be fulfilled by any person are not assigned in advance but are delegated dynamically throughout the surgery, depending on the circumstances. Some options for the task distribution in view of the anticipated challenges may be contemplated before surgery. However, if unforeseen complications arise, new arrangements are conceived and instituted at the time.

A specific reason tasks may be redistributed during surgery is that problems emerge for which a team member does not possess the necessary skills. Thus a senior physician may take over a step of the procedure initially assigned to someone else. Another possibility is that the procedure itself may need to be altered because of the specific problems encountered, such that the steps of the revised procedure must be reassigned among team members. Team members will also assist their colleagues to balance the workload within the group. An anesthetist, for example, may help the scrub nurse if the circulating nurse is busy. Lastly, the task distribution may change as a result of additional resources being mobilized for the task at hand. For instance, due to unforeseen complications during surgery, it may be necessary to call a more experienced clinician for help. According to Bogdanovic et al. (2015), such open-ended fine tuning of the task distribution, including the temporary assistance provided by team members across their professional demarcations, provides the flexibility necessary for dealing with situational variability, minimizes pressure on the team, and enables a smoothly running procedure.

### Necessity of integrated system design

The preceding discussion has clear implications for system design. First, designing for adaptation is essential so that workers can handle a wide variety of events, including both routine and novel ones, effectively. Moreover, workers must be supported in adapting both their behavior and structure, effortlessly and seamlessly. It is important to recognize that changes in behavior may or may not be associated with changes in structure. In addition, changes in structure may be associated with behavioral opportunities not available to workers otherwise. Irrespective of these fine distinctions, designing for adaptation must encompass the behavioral and structural possibilities comprehensively if we are to create systems that are resilient in the face of instability and uncertainty.

Evidently, systems are comprised of multiple elements, which must work together in concert in view of a common purpose. Consequently, the aforementioned objectives cannot be achieved by focusing on the design of individual elements, such as the interfaces, teams, training, or automation. In the context of promoting worker adaptation, the need for integrated system design was emphasized by Vicente (2002). He observes that designing for adaptation cannot be achieved in a piecemeal fashion. That is, a system will not necessarily be adaptive simply because it has an ecological interface, even though such interfaces are intended to support adaptation (Rasmussen and Vicente, 1989; Vicente and Rasmussen, 1990, 1992). Instead, to create systems that can adapt successfully, all of the different elements must be designed in a coordinated manner based on a common philosophy, specifically a philosophy focused on promoting adaptation. Naikar (2012) echoes these observations, recognizing in particular that a system will not necessarily be adaptive solely on the basis of its team design, even if that is intended to engender flexibility (Naikar et al., 2003). In this paper, we elaborate on these ideas by taking into account the empirical observations described above.

To create adaptive systems, the design of multiple elements must be integrated based on a common philosophy that promotes both structural and behavioral adaptation. It is also clear that to preserve a system's inherent capacity for adaptation to novelty, the designs of the different elements must support the full range of opportunities for structural and behavioral adaptation in the workplace and that they must do so uniformly across multiple actors in the system. Thus, if a team design supports possibilities for structural or behavioral adaptation that an interface design does not, the design of the two elements would not be integrated, or compatible, with respect to the goal of promoting adaptation. Similarly, if an interface design for an actor or group of actors in a system supports possibilities for adaptation that are not recognized or accommodated by the interface designs for other actors in the system, such that some or all of the possibilities cannot be realized by any of the actors, the design of this element would not be integrated across multiple actors in the system. Such approaches would not necessarily foster successful performance in the event of change or novelty, and they might even inhibit it. Moreover, as demonstrated later, simply approaching the design of multiple elements concurrently with the philosophy of promoting worker adaptation may be insufficient to achieve this level of integration. Rather, the design framework must encompass explicit mechanisms for binding or anchoring the designs of multiple elements, so that the system design supports the range of possibilities for adaptation in structure and behavior, across multiple actors, in a coherent fashion.

## Work analysis and design

Designing for adaptation requires special approaches for work analysis, as the way in which the work demands of a system are understood is tightly integrated with how those work demands are supported through design. As is well established now, work analysis techniques may be differentiated on the basis of whether they are normative, descriptive, or formative in orientation (Rasmussen, 1997; Vicente, 1999). The following discussion demonstrates briefly that normative approaches are unsuitable for designing for adaptation, whereas descriptive approaches are insufficient. Instead, a formative approach is necessary.

Normative approaches, such as task analysis techniques that define sequences or timelines of tasks (Kirwan and Ainsworth, 1992), are concerned with specifying the ideal ways in which to perform work under particular conditions. However, in open systems, which are subject to situational variability, the anticipated conditions may never match the conditions that are experienced precisely, such that the recommended task sequences or procedures may not in fact be the most productive or safest way of handling the situation. Moreover, removing autonomy from workers in deciding the best way of performing a task or in following their individual preferences when it is appropriate to do so may be counterproductive for workers' health and ultimately for organizational productivity.

Descriptive approaches, such as some of those described in Schraagen et al. (2000), are concerned with developing a faithful understanding of the cognitive challenges that workers experience in their jobs and the cognitive strategies they employ for dealing with these challenges. On this basis, designs can be developed that support workers in handling these challenges more effectively and that accommodate the variability in work practices observed in everyday work. One limitation of such approaches, however, is that the resulting appreciation of cognitive challenges and viable cognitive strategies is generally constrained to familiar, recurring, or anticipated conditions, which can be studied or observed. The capacity of such approaches to support adaptation to unforeseen events, then, is limited to the extent to which the existing challenges and strategies are relevant to the novel conditions. Descriptive techniques, therefore, must be complemented with a formative approach to work analysis, and CWA offers a suitable starting point.

### Cognitive work analysis

**Figure 1:**
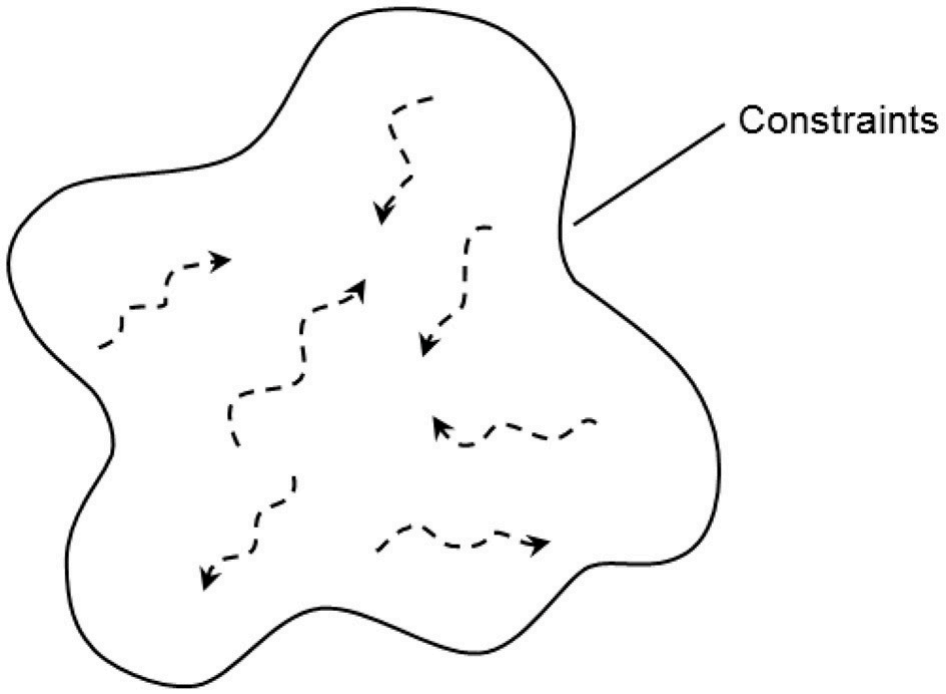
**Within the constraints on successful performance, actors have many possibilities for action**.

CWA is a comprehensive framework for modeling the work demands on actors in terms of the constraints, or boundaries, that must be upheld by their actions irrespective of the particular conditions they are faced with (Rasmussen, 1986; Rasmussen et al., 1994; Vicente, 1999). Thus this framework is concerned with the constraints that are applicable not only in familiar, recurring, and anticipated situations but also in situations that cannot be predicted a priori. Although these constraints must be observed or respected for effective performance, such that they bound the possibilities for action available to actors, within these constraints actors still have many degrees of freedom for action, as indicated by the trajectories in Figure 1. Therefore, using this framework, designs can be developed that deliberately provide actors with the flexibility to adapt their work practices to a wide range of situations without crossing the boundaries of successful performance. In contrast to normative and descriptive approaches, then, which focus on specifying how work *should be done ideally* or *is done currently* in a system, CWA is a formative approach that is concerned with specifying the constraints that bound how work *can be done effectively*.

The CWA framework comprises five dimensions, which are concerned with different types of constraints (Table 1). These dimensions collectively define a constraint-based space, such as that illustrated in Figure 1, in relation to the system of interest. As shown in Table 1, each CWA dimension has special modeling tools for capturing and representing the various constraints on actors. In the current CWA framework, the social organization and cooperation dimension takes advantage of the modeling tools from the preceding dimensions (Rasmussen et al., 1994; Vicente, 1999). However, in this paper the diagram of work organization possibilities (WOP) is introduced as a special modeling tool for this analysis.

**Table 1:** **CWA: Dimensions, constraints, and modeling tools**.

**Dimensions**	**Constraints**	**Modeling Tools**
Work domain analysis	Work domain—constraints placed on actors by the physical, social, and cultural environment, including the system's purposes, values and priorities, functions, and physical resources	Abstraction-decomposition space, abstraction hierarchy (Rasmussen et al., 1994; Vicente, 1999; Naikar, 2013)
Activity analysis	Activity—constraints placed on actors by the activities necessary in the system to achieve the system's purposes, values and priorities, and functions with the available resources	Contextual activity template (Naikar et al., 2006), decision ladder (Rasmussen et al., 1994; Vicente, 1999)
Strategies analysis	Strategies—constraints placed on actors by the cognitive strategies that can be utilized for achieving the activities necessary in the system	Information flow map (Rasmussen et al., 1994; Vicente, 1999)
Social organization and cooperation analysis	Work organization—constraints placed on actors by the ways in which work can be allocated, distributed, and coordinated in the system	Diagram of work organization possibilities
Worker competencies analysis	Workers—constraints placed on actors by the ways in which the work demands of the system can be met given human cognitive capabilities and limitations	Skills, rules, and knowledge taxonomy (Rasmussen et al., 1994; Vicente, 1999)

### Value of cognitive work analysis for design

Considerable empirical evidence exists for the value of CWA for design, specifically in relation to ecological interface design, a framework that utilizes CWA as a basis for designing interfaces for workers in complex sociotechnical systems (Rasmussen and Vicente, 1989; Vicente and Rasmussen, 1990, 1992). For example, as documented in existing reviews (Vicente, 2002; Naikar, 2012), controlled experiments have demonstrated the value of ecological interface design for process control (Christoffersen et al., 1996; Pawlak and Vicente, 1996; Reising and Sanderson, 1998, 2000a,b; Ham and Yoon, 2001; Jamieson, 2007; Lau et al., 2008), information retrieval (Xu et al., 1999), neonatal intensive care (Sharp and Helmicki, 1998), network management (Burns et al., 2003), aviation (Borst et al., 2006), and military command, and control (Bennett et al., 2008). Collectively, the results of these studies demonstrate that ecological interface design can be applied to a range of systems and that, for those systems, this framework can uncover novel information requirements that can lead to better performance by workers in comparison with that obtained with existing interfaces.

The value of CWA for problems other than interface design has also been demonstrated. Detailed industrial case studies have shown, for example, that CWA can be used for selecting system designs (Naikar and Sanderson, 2001), designing teams (Naikar et al., 2003), and developing training systems (Naikar and Sanderson, 1999) that promote flexibility. As these applications of CWA were executed in industrial settings, experimental investigations were unfeasible. However, the value of CWA for these applications was demonstrated on the basis of its ability to impact practice, its uniqueness in comparison with the design outcomes obtainable with conventional approaches, and its feasibility of implementation within a project's schedule, personnel, and financial resources (Naikar, 2013). These criteria are more commonly applied for assessing worth in industrial practice (Whitefield et al., 1991; Czaja, 1997; Vicente, 1999).

### Limitations of cognitive work analysis for design

**Figure 2:**
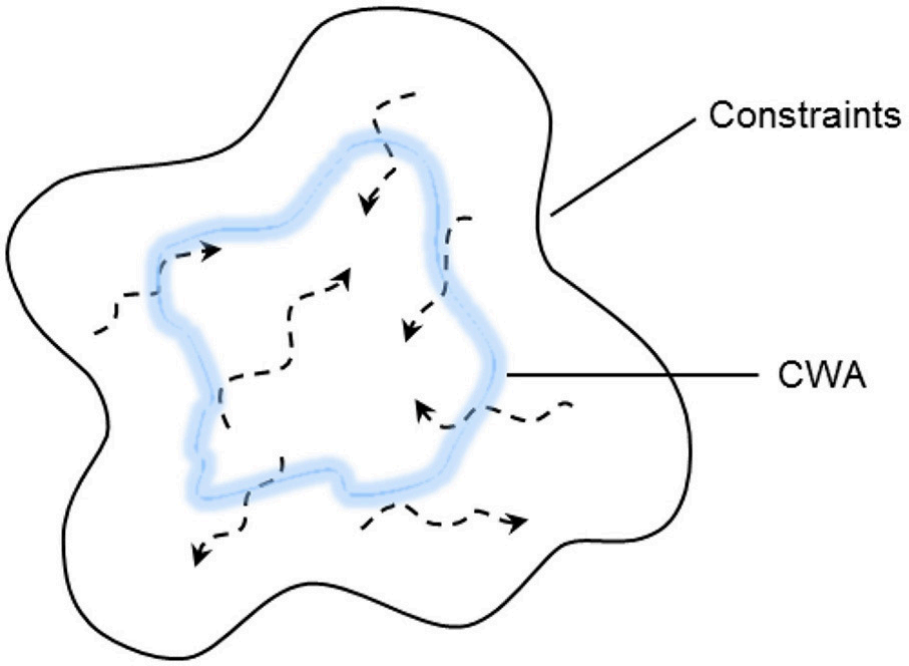
**CWA supports adaptation but limits the possibilities for action available to workers, thus restricting a system's inherent capacity for adaptation**.

While it is clear that CWA can support adaptation, in this paper we observe that this framework has two, related, limitations that could restrict a system's inherent capacity for adaptation (Figure 2). The first has to do with the capacity of this framework to support adaptations in the work organization, or structural adaptation. The second concerns its capacity to facilitate the integration of multiple system elements to produce an integrated system design.

One reason that CWA is limited in its capacity to promote adaptation is that although this framework can support actors in adapting their behavior, in its current form it does not necessarily support actors in adapting their structure, especially in unforeseen situations. Yet, as the empirical studies described earlier in this paper and elsewhere show, adaptations in the work organization are also critical for successful performance. The fundamental texts on CWA by Rasmussen et al. (1994) and Vicente (1999) do recognize that complex sociotechnical systems are characterized by flexible organizational structures, such that the structures actors adopt may vary subtly or significantly in response to the local context. Thus they point out that the social organization and cooperation dimension of CWA must be concerned with the various organizational structures that are relevant. Moreover, the texts observe that shifts in structure are governed by such criteria as the competencies of actors, the access actors have to information or the means for action, the requirements for safety and reliability, the need for compliance with policies and regulations, the requirements for workload sharing, and the need for minimizing coordination demands. However, neither text offers a formative approach for analyzing the work organization. Instead, the suggested approach seems descriptive in orientation as it appears to be concerned with organizational structures that can be observed or are judged to be reasonable in recurring classes of situation (Naikar and Elix, 2016a).

**Figure 3:**
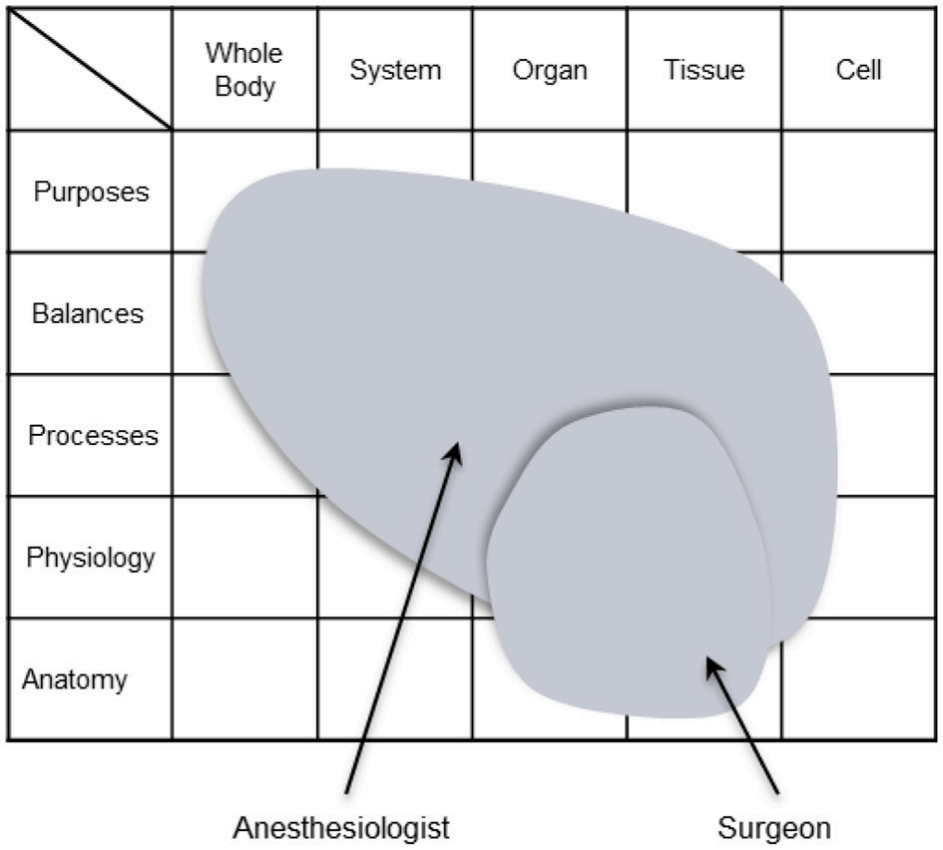
**Vicente's (1999) use of the abstraction-decomposition space to illustrate the distribution of work demands across a surgeon and an anesthesiologist during surgery**. Reprinted with permission of Lawrence Erlbaum Associates.

As a case in point, Vicente (1999) discusses that, within the CWA framework, the analysis of organizational structures is undertaken in relation to particular classes of situation and, to illustrate this approach, he provides an example of how CWA can be used to analyze the organizational structures in a health care system. Specifically, he describes how the work demands of surgery may be distributed differently across a surgeon and an anesthesiologist, and he points out that the distributions of work demands may change if the patient is in pre-operation rather than in surgery. Furthermore, to complement his discussion, he illustrates how models from the CWA framework may be used for representing such distributions (Figure 3). However, in this approach, CWA is being used to describe the organizational structures that are adopted by workers in recurring classes of situation, rather than to understand the structures that can be adopted irrespective of the situation. This approach may be useful for developing designs that support workers in commonly occurring situations, which is important. However, designs based on this approach may not be suitable for dealing with some kinds of situational variability or with unanticipated events particularly, because they may not support the organizational structures that are relevant—or that emerge—in unforeseen circumstances. Moreover, as these structures may present new behavioral opportunities, the resulting designs may not support some behavioral possibilities.

Another, related, reason that CWA is limited in its capacity to facilitate adaptation concerns its ability to support integrated system design, whereby the design of multiple elements are coordinated across multiple actors in the system, such that workers are supported in adopting the range of possibilities for structural and behavioral adaptation in a unified manner. As discussed in more detail in the next section, to facilitate the integration of multiple elements in a way that promotes adaptation, the design of each element must be anchored to a common set of constraints. In complex sociotechnical systems, which are comprised of multiple actors, the full set of constraints that is relevant to each actor, or group of actors, in the system is dependent on the organizational structures that are possible. Accordingly, the design of each element must be coordinated around the organizational constraints. Hence the lack of a formative means for analyzing the organizational structures that are relevant, irrespective of the situation, does not limit simply the capacity of CWA to promote structural adaptation but also its capacity to facilitate the integration of multiple elements, across multiple actors, to produce an integrated system design.

We do not suggest here that a formative analysis of the work organization is sufficient for creating an integrated system design. It is also important, for example, to have systematic processes for respecting the organizational constraints in the design of each element, as discussed in more depth later. The formative analysis of organizational structures, however, is a central step in creating an integrated system design. Perhaps it is also worth making the point explicitly that a formative analysis of the work organization in itself does not guarantee that multiple elements will be considered in the design process, but, once again, this analysis is essential for the designs of multiple elements to be well integrated, as elaborated in the next section.

Finally, it is worth noting that existing design approaches based on CWA are limited in their capacity to promote adaptation in the manner concerned with here. First, detailed design approaches are focused largely on individual system elements, such as the interfaces (Rasmussen and Vicente, 1989; Vicente and Rasmussen, 1990, 1992) or teams (Naikar et al., 2003; Naikar, 2013), although this is not to say that the need for integration with other elements was unappreciated. In relation to system design, Vicente (1999) makes the observation that particular phases of CWA can be used to inform particular classes of system design interventions. For example, he discusses that work domain analysis can be used to inform the design of information systems, that social organization and cooperation analysis can be used to inform the design of teams, and that worker competencies analysis can be used to inform the design of training programs. However, it is unclear how Vicente (1999, 2002) intended the designs of the different elements to be integrated (Naikar and Elix, 2016a). If the designs of these elements are informed by different phases of CWA, such that they are based on distinct sets of constraints, the resulting designs would not necessarily support the same possibilities for adaptation. Alternatively, if the design of each element is based on all five phases of CWA, the resulting designs may be integrated but only in relation to a reduced space of possibilities for action, as the analysis would be restricted deliberately to organizational structures that can be observed or are judged to be reasonable in recurring classes of situation.

Further to Vicente (1999, 2002), some approaches have addressed how particular phases of CWA can be used to support different stages of the system lifecycle, such as requirements definition, design, and evaluation, and to support the design of a variety of system elements, such as the interfaces, teams, and training (Sanderson et al., 1999; Hori et al., 2001; Read et al., 2015a,b). It would be fair to say that all of these approaches recognize at some level the need for the design of multiple elements to be integrated in some fashion, although Hori et al. (2001) and Sanderson et al. (1999) do not address this point explicitly. Read et al. (2015b) discuss the need to ensure that the design of all of the elements are coordinated and, in the context of a case study, Read et al. (2015a) describe the use of a template for summarizing a design concept, which requires that design features associated with all system elements are documented. On the basis of the information provided in these papers, it seems that this process could help to ensure that the designs of multiple elements are considered concurrently, although from the case study it appears that this is not a guaranteed result, given the ratings of the four participants in the design process and the analyst's reflections. In any case, assuming all elements are considered concurrently, it is unclear in what way, or on what basis, the design of the different elements would be coordinated using the process described, and thus what manner of integration the process would promote. However, considering that the process is based on the existing CWA framework, one can assume that it would be limited in its capacity to support structural adaptation and to facilitate the integration of multiple system elements in the fashion with which this paper is concerned.

## Integrated system design

This paper proposes an approach for integrated system design, based on extensions of CWA. The approach develops substantially ideas described initially by Naikar (2006, 2012, 2013) for the analysis of the work organization and by Naikar and Elix (2015) for coordinating the design of multiple system elements. The express intent of this approach is to promote the capacity of sociotechnical systems for adaptation.

**Figure 4:**
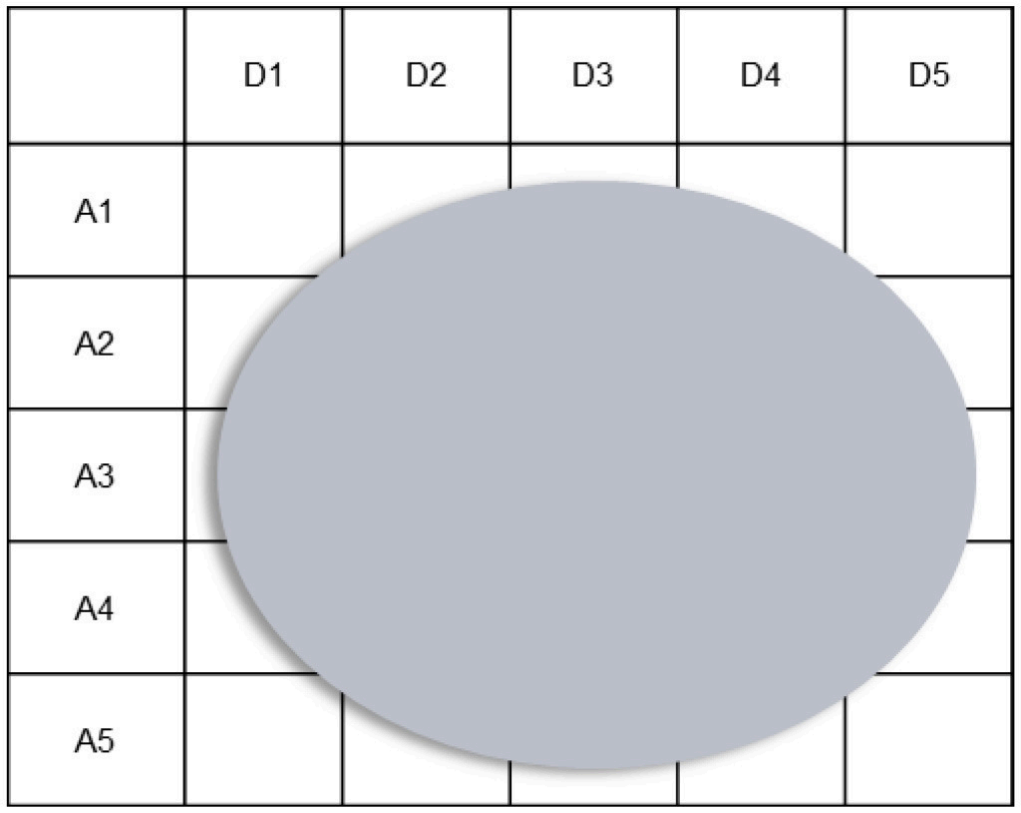
**Use of the abstraction-decomposition space to emphasize that a single actor could not possibly attend to all of a system's work demands**. “A” signifies a level of abstraction and “D” signifies a level of decomposition.

The proposed approach has two particular premises. First, the approach presupposes that complex sociotechnical systems are comprised of multiple actors, as a single actor could not possibly attend to all of a system's work demands (Figure 4). For example, a single actor could not possess or develop the full set of knowledge and skills necessary for dealing with all of the system's work demands effectively. Similarly, a single actor could not have the physical and mental capacity to cope with all of the system's work demands in the combinations and pace at which they occur. The significance of this straightforward assumption is made clear later.

Another premise of the proposed approach is that in complex sociotechnical systems there is usually no single or best way of organizing work, or of distributing the work demands across multiple actors. Instead, as empirical studies such as those cited earlier (Rochlin et al., 1987; Bigley and Roberts, 2001; Bogdanovic et al., 2015) show, flexible work structures that can be adapted to local contingencies are necessary for dealing with the demands of a range of situations, including unforeseen events. This means, then, that designs must support actors in adapting not only their behavior but also their structure, such that it is possible for actors to meet the demands of a variety of circumstances, some of which may be completely novel to them.

In line with these premises, the proposed approach for integrated system design recognizes that to promote the capacity of sociotechnical systems for adaptation, it is necessary to understand the set of possibilities for work organization in a system irrespective of the situation. From a design perspective, this is necessary not simply for supporting multiple actors in adapting their structure but for coordinating the design of multiple elements, such as the interfaces, teams, training, and automation. As a result, actors will be supported in adapting their structure as well as their behavior —in a unified fashion—to meet the demands of a range of circumstances. Accordingly, the approach places emphasis on demarcating the set of possibilities for work organization in a system, given the system's constraints, and subsequently developing designs for each element that can accommodate the range of possibilities. These ideas are elaborated in the following discussion.

For the purposes of integrated system design, the set of possibilities for work organization in a system is delineated through extensions of CWA, rather than any other work analysis technique, as a formative approach is necessary for supporting adaptations in both behavior and structure across a range of situations. As demonstrated in detail later, the possibilities can be delineated within the social organization and cooperation dimension of CWA (Table 1) by applying the criteria that govern shifts in work organization in a formative manner to examine how the work demands of the system can be distributed across actors—both human and automata. Ideally, the work demands would be derived from the first three dimensions of CWA, namely work domain analysis, activity analysis, and strategies analysis. However, given practical considerations, the work demands may be derived solely from work domain analysis, as it encompasses both novel and anticipated situations (Naikar and Elix, 2015, 2016a). Once the organizational possibilities have been defined, designs for each of the system elements can be developed to support those possibilities at the three levels of cognitive control that actors can bring to the performance of a task. These three levels of cognitive control, skill-based, rule-based, and knowledge-based behavior, are considered within the worker competencies dimension of CWA. Thus the proposed approach coordinates the design of multiple system elements around the organizational constraints.

**Figure 5:**
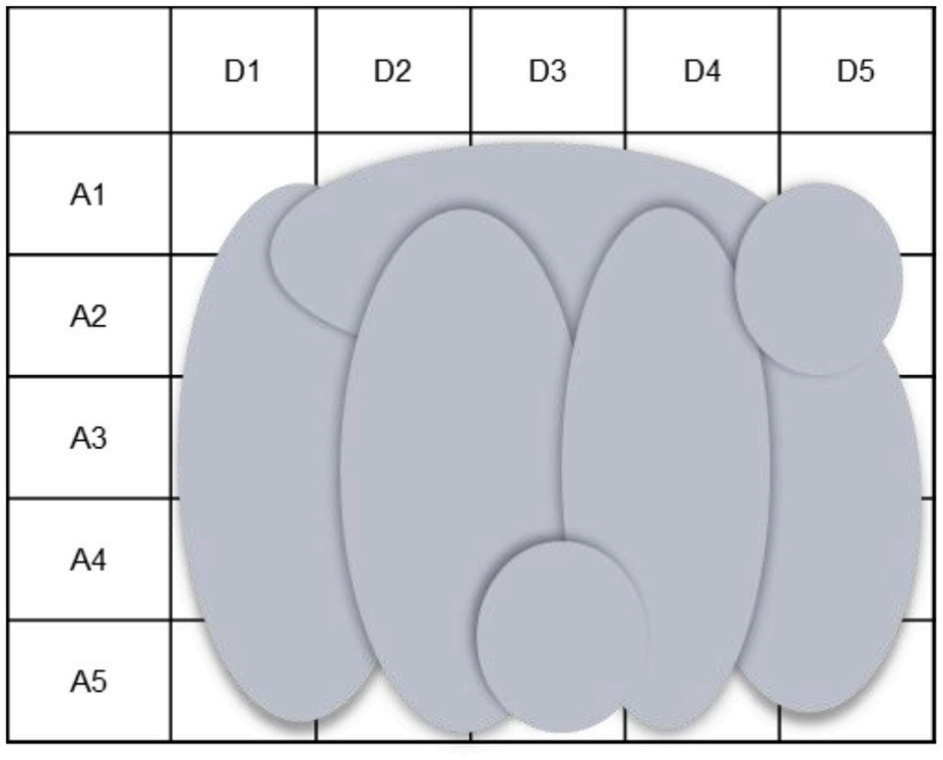
**Use of the abstraction-decomposition space to illustrate that when there are multiple actors, the constraints that are relevant to an actor, or group of actors, are dependent on the possibilities for work organization**. “A” signifies a level of abstraction whereas “D” signifies a level of decomposition.

The set of possibilities for work organization in a system is regarded as the central mechanism for integrating the design of multiple elements because complex sociotechnical systems are comprised of multiple actors. To create an integrated system design, one in which all of the elements support adaptation in a coherent fashion across multiple actors, the design of each element must be anchored to a common set of constraints. Given multiple actors, the constraints of the work domain, activity, strategies, and workers that are applicable to an actor, or group of actors, are dependent on the possibilities for work organization (Figure 5). Hence the design of each element, for each actor, must be coordinated around these possibilities, or organizational constraints. While the design of each element must also respect the constraints of the work domain, activity, strategies, and workers, the designs of these elements can only be coordinated around those constraints if it is assumed that a single actor is responsible for all of the system's work demands. However, this design approach is unsuitable for complex sociotechnical systems, as multiple actors are necessary for fulfilling the system's work demands.

Notably, as the constraints that are relevant to a particular actor or group of actors are dependent on the possibilities for work organization, understanding the set of possibilities is essential not only for supporting actors in adapting their structure but also in adapting their behavior. As indicated earlier, the different structural possibilities are associated with distinct behavioral opportunities. Therefore, to appreciate the full set of behavioral possibilities available to particular actors, it is necessary to establish the full set of work structures in which they can participate. Otherwise, the resulting constraint-based space for each actor will be smaller than their actual space of possibilities for action. This means that the associated designs, though offering some degree of flexibility to each actor, will limit the possibilities for action available to them, ultimately restricting the capacity of the system for adaptation.

By emphasizing the necessity of defining the set of possibilities for work organization independently of the situation, the proposed approach promotes greater adaptation than can be achieved by focusing designs on a subset of possibilities. For example, the approach can lead to designs that support greater adaptation than designs based on work structures observed in recurring situations. Similarly, it can lead to designs that promote greater adaptation than those based on work structures deemed ideal under certain conditions. This approach, then, can foster the development of more robust or resilient systems that are capable of coping with idiosyncratic circumstances or situations involving small variations from recurring or pre-defined conditions, as even small changes in context can require adaptation by workers. Moreover, it can foster the development of systems with greater capacity to deal with novel events, which is particularly important given that these events are widely regarded as posing the most significant threats to performance (Rasmussen, 1968a,b, 1969, 1986; Perrow, 1984; Reason, 1990; Rasmussen et al., 1994; Leveson, 1995; Vicente, 1999).

The proposed approach therefore enhances the quality of the integration of multiple system elements, with respect to the goal of promoting adaptation, compared with that achievable by designing the various elements using existing design approaches based on CWA. As an illustration, the application of existing approaches to design particular elements could involve using the ecological interface design framework (Rasmussen and Vicente, 1989; Vicente and Rasmussen, 1990, 1992) to create the displays for a system and a technique described by Naikar et al. (2003; also see Naikar, 2013) to develop the team designs for that system. However, applying these techniques in combination would not necessarily ensure that the designs of the two elements are well coordinated, particularly because there is no explicit mechanism for tying together, or binding, the designs of the interfaces and teams across multiple actors in the system.

In particular, the ecological interface design framework cited above is based on the constraints of the work domain and workers, whereas the team design approach is concerned with the constraints of the work domain and activity. Notably, Bennett and Flach (2011) describe an approach for ecological interface design that incorporates the constraints of the work domain, activity, and workers. Nevertheless, even if the designs of both elements were anchored somehow to a common set of constraints, whether this is the constraints of the work domain, activity, workers, or all of these constraints, this approach would be insufficient for complex sociotechnical systems.

Assuming that the existing techniques for both elements involve some kind of recognition, formal or informal, of there being multiple actors and of there being different ways of organizing work among these actors, as the team design technique does at least, the resulting designs would most probably take into account only a subset of the work organization possibilities, say those that can be observed or anticipated. Consequently, while the designs of the two elements may be integrated across multiple actors in the system, by anchoring the designs of both elements to the constraints considered relevant to each actor or group of actors, the designs would be integrated only in relation to a reduced space of possibilities for adaptation. Such an approach would restrict the system's inherent capacity for adaptation.

The proposed approach for integrated system design, then, has implications for existing design approaches based on CWA. Irrespective of which element or elements are of concern, it is necessary to incorporate the set of work organization possibilities in the designs of those elements. Thus, relative to existing approaches, the proposed approach would enhance the capacity of the system for adaptation by promoting structural adaptation, providing opportunities for behavioral adaptation associated with the structural possibilities, and facilitating the integration of multiple elements, such that the overall design preserves the system's underlying capacity for adaptation, across multiple actors, in a systematic fashion.

In summary, the proposed approach can be considered integrative on two levels. First, it provides a unified means for supporting adaptations in both behavior and structure. Thus, even if the focus is on an individual element, by incorporating the constraints on the possibilities for work organization in the design of that element, alongside the other constraints, the resulting design would support adaptations in both behavior and structure. Second, the approach provides a lynchpin—in the form of a common set of work organization possibilities—for integrating the design of multiple elements. This mechanism is important because simply incorporating these possibilities into the design of a single element would be conducive to supporting adaptation but insufficient. Rather, the designs of the various elements must be coordinated, across multiple actors in the system, such that the system design supports the range of possibilities for structural and behavioral adaptation in a coherent manner.

### Analysis

In creating an integrated system design, then, the set of work organization possibilities is a central concept in the analysis and design effort. Thus this section shows how the set of work organization possibilities may be defined, while the next section shows how these possibilities may be utilized in design.

The precise aim of the analysis phase is to demarcate the set of possibilities for work organization in a system irrespective of the situation. Thus the possibilities must be defined in a formative manner, such that they are not limited to particular conditions but are relevant to any situation, even those that cannot be anticipated. Consequently, designs can be developed to support worker adaptation to a variety of conditions, including novel events. The key question then is how the set of all possible work structures in a system may be identified without consideration of the full set of circumstances in which they may be implemented, as all of these circumstances cannot be predicted a priori.

**Figure 6:**
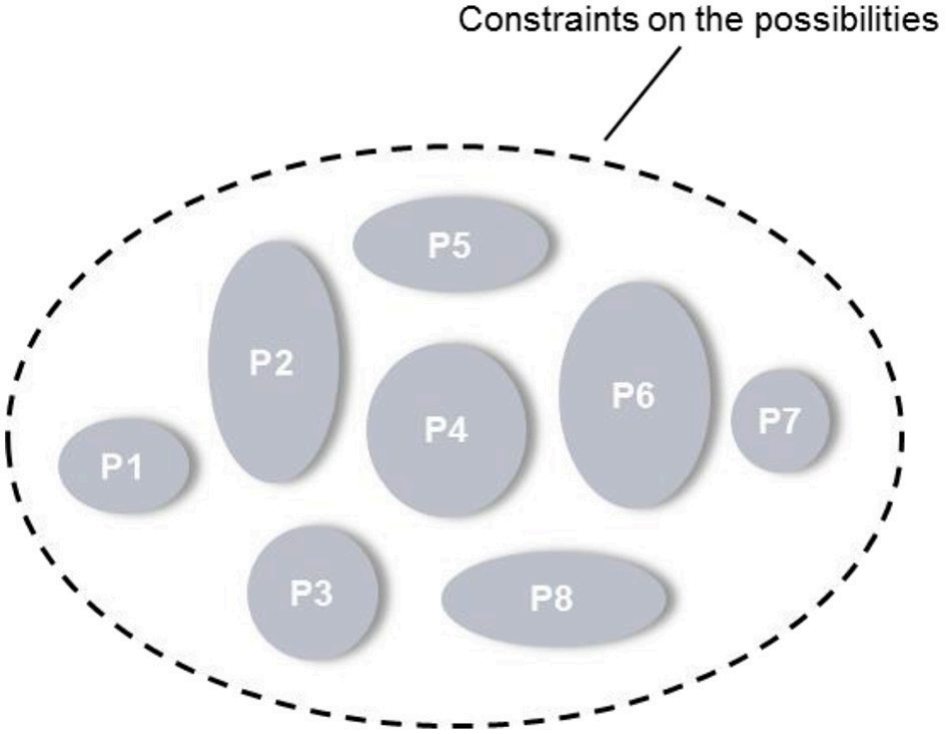
**The set of possibilities for work organization is delineated by defining the constraints on the possibilities**. “P” denotes a work organization possibility.

The essence of the approach is encapsulated in Figure 6. Basically, this figure shows that the set of possibilities for work organization in a system can be delineated independently of the situation by defining the constraints on the possibilities, rather than describing the possibilities themselves. As will be demonstrated in the following discussion, these constraints can be identified by analyzing the *limits* placed on the distribution of *work demands* across actors by the *criteria* that govern shifts in work organization, as these criteria will constrain the structures actors can adopt.

It is important to appreciate that the criteria that dynamically govern shifts in work organization exclude certain work structures from consideration altogether. This point is not recognized explicitly by either Rasmussen et al. (1994) or Vicente (1999). Depending on the access actors have to information or controls, for instance, only certain ways of distributing the work demands across actors will be possible in the system regardless of the situation. Likewise, based on organizational policies or the competencies of actors, only particular work arrangements will be permissible or feasible at any point in time. Thus the criteria exclude certain work structures outright, as well as constraining the structures that are suitable under particular conditions, thereby dynamically governing shifts in work organization. Consequently, by amalgamating the criteria with the work demands of the system to identify the structures that are to be excluded altogether, the set of possibilities for work organization in the system may be circumscribed.

**Figure 7:**
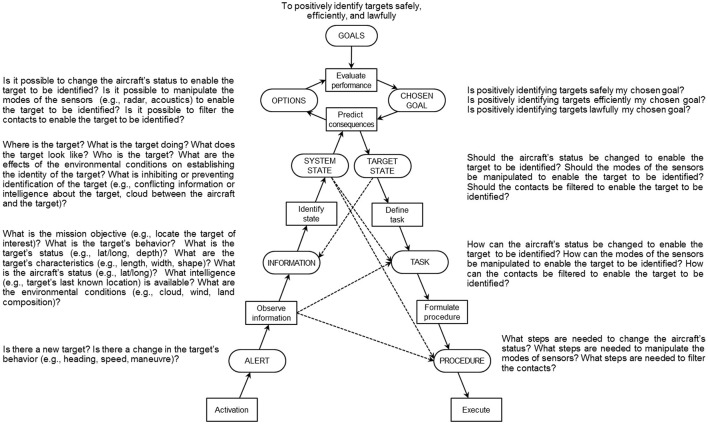
**A modified decision ladder identifying some of the work demands of a future maritime surveillance aircraft**.

In an idealized implementation of the approach, then, the first step is to define the work demands of the system with the first three dimensions of CWA, namely work domain analysis, activity analysis, and strategies analysis, consistent with a constraint-based perspective. Accordingly, the work demands of the system will be captured in the form of an abstraction-decomposition space or abstraction hierarchy, a contextual activity template, a set of decision ladders, and a set of information flow maps (Table 1). As an illustration, Figure 7 presents a modified decision ladder from a set of eight that resulted from an activity analysis of the Royal Australian Air Force's future maritime surveillance aircraft (Elix and Naikar, 2008). This model represents some of the decision-making demands associated with identifying targets, such as an enemy submarine, from the aircraft. For example, the work demands involve positioning the aircraft and manipulating its various sensors to obtain certain information about the target, such as its location and characteristics, so that the target's identity can be established, even in the face of such obstacles as the environmental conditions. The basic elements of the decision ladder template are described in detail by Rasmussen et al. (1994), Vicente (1999), and Naikar et al. (2006).

Subsequently, in the social organization and cooperation dimension, the work organization criteria are applied to the work demands to demarcate the set of possibilities for work organization in the system. As indicated above, this process involves examining the limits placed on the allocation or distribution of work demands across actors by each of the criteria, irrespective of the situation. In this paper, the same six criteria observed by Rasmussen et al. (1994) and Vicente (1999) to dynamically govern shifts in work organization are utilized. In studies of two military systems, an Airborne Early Warning and Control aircraft (Naikar et al., 2003; Naikar, 2013) and the future maritime surveillance aircraft referred to earlier, no additional criteria were identified. However, it is possible that other criteria may be relevant for different systems.

The limits on the possibilities for work organization can be identified by considering the following kinds of question in relation to the work demands captured in the various CWA models:

- *Compliance*: Does the need for compliance with policies or regulations constrain how the work demands can be allocated or distributed across actors?
- *Safety and reliability*: Does the need for safety or reliability place constraints on the allocation or distribution of work demands?
- *Access to information/controls*: Does the access actors have to information or controls constrain the allocation or distribution of work demands?
- *Coordination*: Does the need for feasible coordination requirements place constraints on how the work demands can be allocated or distributed?
- *Competencies:* Does the need for feasible competency requirements constrain the allocation or distribution of work demands?
- *Workload*: Does the need for manageable workload constrain how the work demands can be allocated or distributed across actors?

**Figure 8:**
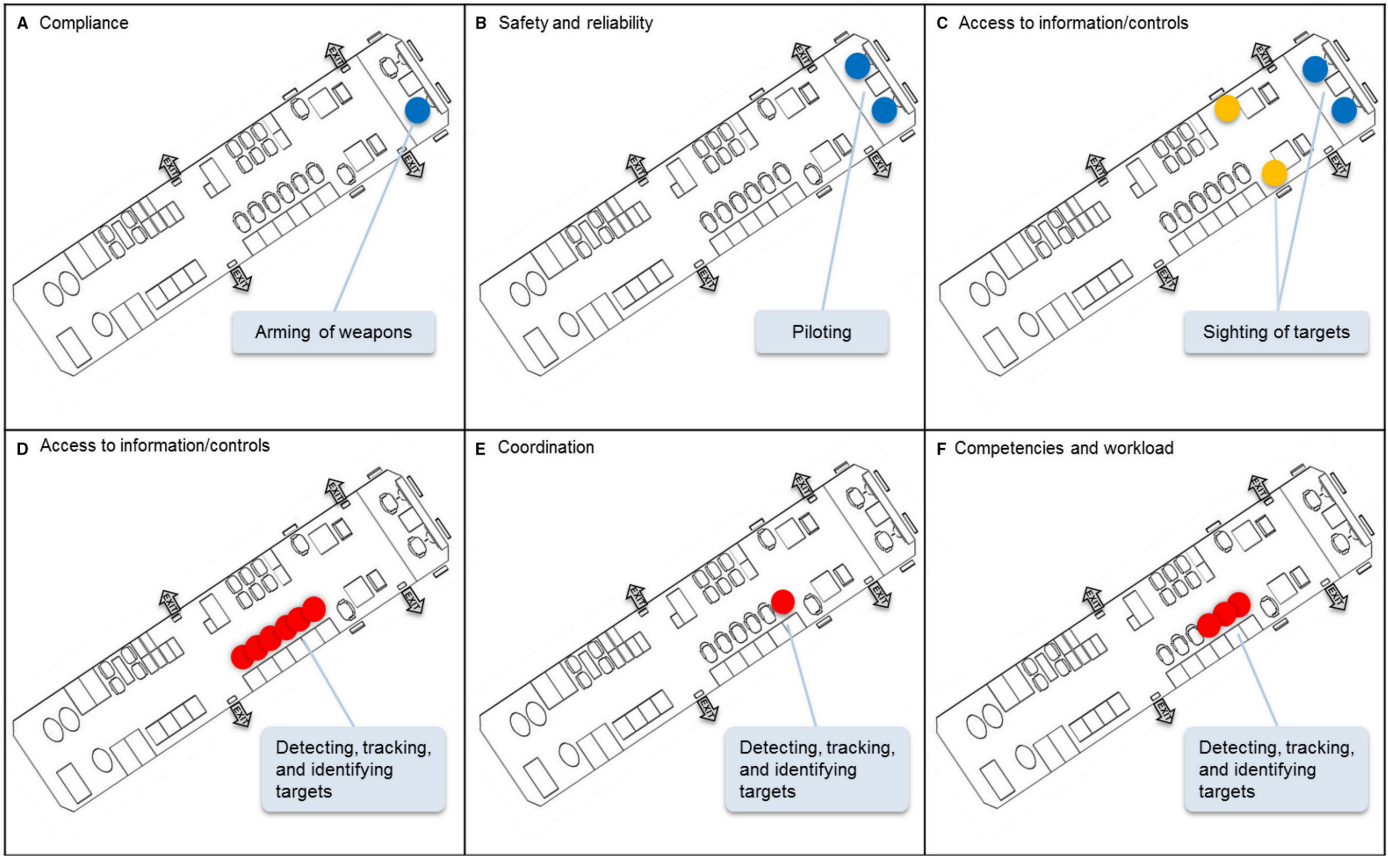
**Illustration of the application of the work organization criteria to a future maritime surveillance aircraft**. **(A)** Compliance; **(B)** Safety and reliability; **(C)** Access to information/controls; **(D)** Access to information/controls; **(E)** Coordination; **(F)** Competencies and workload.

For example, in the case of the maritime surveillance aircraft, the need for compliance with organizational regulations constrains the captaincy of the aircraft to one of the flying crew rather than tactical crew. Therefore any work demand requiring the authority of the captain, such as the arming of weapons, must be allocated to one of the flying crew (Figure 8A). Furthermore, the safety and reliability criterion constrains the responsibility of piloting the aircraft to two people, even though a single person would have the capacity to handle this responsibility. Consequently any work demand associated with piloting the aircraft must be allocated to at least two actors (Figure 8B). Third, the criterion of access to information or controls constrains the allocation of any work demand requiring a window, such as the sighting of targets, to actors in the flight deck or at observer stations in the cabin (Figure 8C). In addition, this criterion constrains the control of four sensor systems (i.e., the radar, electro-optical/infrared, electronic support measures, and acoustics sensors) for detecting, tracking, and identifying targets to actors at any of six workstations in the cabin (Figure 8D). Finally, while the criterion of minimizing coordination would constrain the operation of all of the sensors to a single actor (Figure 8E), the requirement for crew members to develop the necessary competencies within a reasonable timeframe and have a manageable workload would result in the allocation of these sensors to more than one actor (Figure 8F).

It is important to emphasize that the criteria are applied to the work demands independently of the situation. This means that the limits that are identified on the allocation or distribution of work demands must hold regardless of the circumstances or, in other words, be relevant to any situation. From a practical perspective, then, when analysts step through the process of applying the criteria to the work demands, they are likely to find that while certain possibilities for work organization can be excluded outright on this basis, there are many remaining possibilities and which of these possibilities will be adopted by actors cannot be established independently of the situation.

In some cases, these “ambiguities” may be resolved by analysts in relation to certain classes of situation, such as the work situations in a contextual activity template (Naikar et al., 2006), which may be informative for design but limited in that there is no accounting for unanticipated events or unexpected variations in situations. However, in many cases, these uncertainties can only be resolved by actors in relation to the particularities of a situation, given that these cannot always be predicted a priori. For example, although actors may generally seek to minimize coordination requirements in enacting organizational structures to deal with events, there may be circumstances in which they adopt work structures involving greater coordination because of the workload of particular actors at that point in time. Therefore, often the criterion of coordination will not result in limits on work organization being established conclusively. The same applies to the workload criterion in that there may be times when actors adopt organizational structures involving a high workload for some actors, although they may generally seek a manageable workload for all actors.

Hence, in applying the criteria to the work demands, it is important to focus on those limits that cannot be broken, irrespective of the situation. This means that the boundaries on work organization will stem largely from the criteria of compliance, safety and reliability, access to information and controls, and competencies, as event-independent limits may be derived more readily from these criteria. For instance, the access actors have to some kinds of information or controls will not vary according to situation. Similarly, many organizational regulations will hold across all situations. Nevertheless, despite these constraints, actors will still have many degrees of freedom for action, such that any of the criteria may be invoked online and in real time by actors to enact organizational structures that are suitable given the circumstances. Thus the criteria will still govern shifts in work organization dynamically.

**Figure 9:**
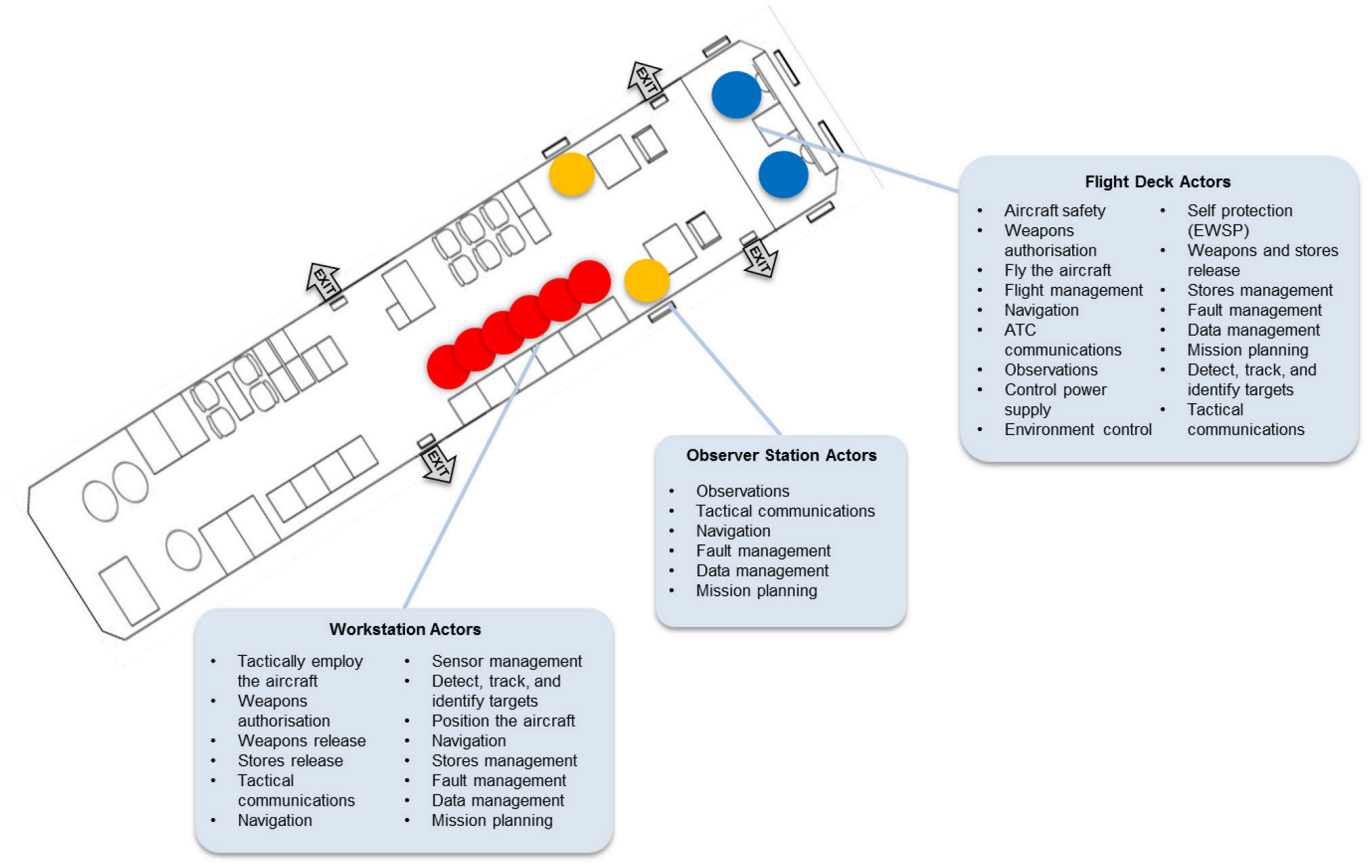
**Modified diagram of work organization possibilities for a future maritime surveillance aircraft**.

Once the criteria have been applied to the work demands to identify the limits on their distribution, it is possible to create a diagram of work organization possibilities for the actors in the system. Figure 9 shows a modified representation of the resulting diagram for the future maritime surveillance aircraft (The full diagram cannot be reproduced here because of space limitations and proprietary restrictions). This figure identifies some of the actors in the system, in terms of their positioning at particular stations on the aircraft, and provides an event-independent representation of the work demands for which these actors can be responsible.

**Figure 10:**
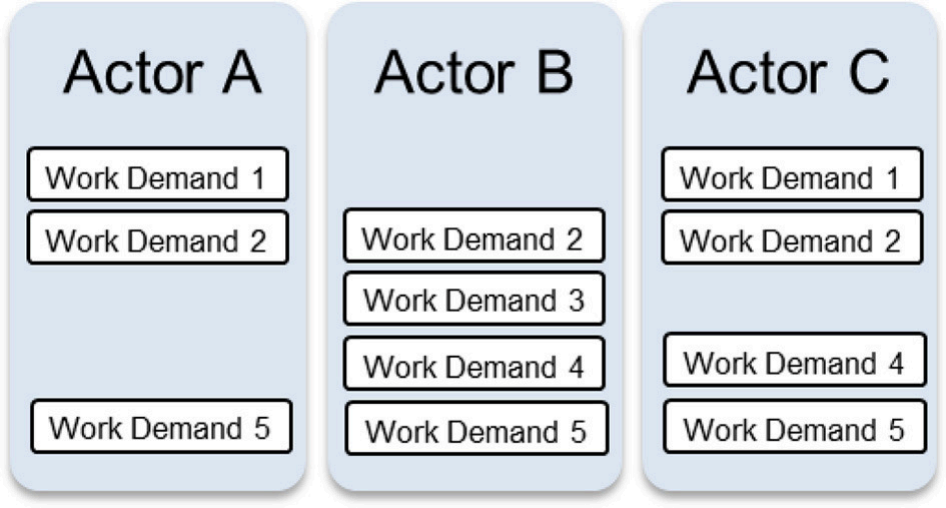
**Generic illustration of the diagram of work organization possibilities**.

For the sake of simplicity, Figure 10 depicts the diagram of work organization possibilities in a generic form. In the following discussion, this figure will be drawn on to highlight some key features of this formative representation. Some examples from the maritime surveillance aircraft will also be provided.

### Can be, not will be

An important feature of the WOP diagram is that it results in an understanding of the set of work demands for which an actor *can* be responsible. Which work demands an actor *will* be responsible for at any point in time is situation-dependent, such that the responsibilities of actors could vary over time. For example, initially Actor A could be responsible for Work Demand 2 but subsequently this responsibility could be assumed by Actors B or C (Figure 10). In the same way, initially Actor B could be responsible for Work Demands 2, 3, 4, and 5 and subsequently for just Work Demand 3.

In the case of the maritime surveillance aircraft, both of the flying crew can take responsibility for the work demands associated with navigating the aircraft (Figure 9). Therefore, the responsibility for these work demands might shift between these actors, depending on the situation, such that at one point in time one of these actors has this responsibility, whereas at another point in time the other actor has this responsibility. Moreover, actors at the observer stations and workstations in the aircraft's cabin can also contribute to some of the navigation work demands, such that the responsibilities for these activities could shift to these actors on certain occasions. In the same way, the actors on the flight deck have access to certain information obtained by the aircraft's sensor systems, so that, when necessary, they can contribute to some of the work demands associated with detecting, tracking, and identifying targets, either alongside or instead of the actors at the six workstations. Finally, each of the actors at the six workstations has access to the information and controls necessary for commanding the mission, which means that the responsibilities for the associated work demands can shift between these actors if required.

### Constraints vs. possibilities

Another feature of the WOP diagram is that it demarcates the set of possibilities for work organization in a system, or the constraints on the possibilities, but it does not portray each possibility. In other words, it depicts the fundamental boundaries on the allocation or distribution of work demands, from which the various possibilities may be derived, but it does not elucidate each possibility. This distinction may be clarified further with a simple example. Figure 10 shows that Actors A and C can take responsibility for Work Demand 1. These are the constraints or boundaries on the possibilities. Given these constraints, the possibilities are: Actor A has this responsibility, Actor C has this responsibility, or Actors A and C share this responsibility. Thus, in a given situation, if the safety criterion is emphasized, for instance, one of these possibilities may be adopted, whereas if priority is given to the criterion of workload sharing, another possibility may be adopted. Which possibility is adopted will depend on the details of the situation, which may not always be known a priori, such that the problem can only be resolved online and in real time by actors.

In the case of the maritime surveillance aircraft, the responsibility for the sighting of targets can be assumed only by actors positioned at a window and thus at four stations on the aircraft—two flight deck stations and two observer stations (Figure 9). These are the constraints on the allocation of this work demand. However, within these constraints, there are numerous possibilities for work organization. If one considers just the two flying crew, the possibilities are that one of the flying crew has this responsibility, the other flying crew has this responsibility, or both flying crew share this responsibility. If one includes the actors at the two observer stations, one at each station, the number of possibilities increases to 15. Moreover, if one considers the fact that each of the four stations could accommodate more than one actor, if necessary, the possibilities are considerably greater. As an example, if there is an electrical failure, such that none of the sensors can be used for detecting targets, more than one actor might be positioned at each of the four stations to increase the chances of finding the target. The WOP diagram accounts for these possibilities but it does not describe each possibility.

### Computable, but unnecessary

Clearly, then, depending on the scale of the system and the level of granularity at which the work demands are modeled, the number of possibilities may be very large. In the case of the future maritime surveillance aircraft, for example, a rough counting revealed the number of possibilities to be in the order of 10^27^. However, while it may not be impossible to compute all of the possibilities, it is unnecessary to do so. That is, to support adaptation, designs must simply take into account the constraints on the possibilities. As long as a design considers the set of work demands for which actors *can* be responsible, actors *will* be able to handle those work demands effectively if and when the need arises. For instance, the interface designs at the various stations on the maritime surveillance aircraft need only accommodate the set of work demands for which actors positioned at those stations can be responsible, as represented in the WOP diagram (Figure 9). As a result, actors will be able to implement any one of the possibilities out of the full set if necessary.

### Emergent, not planned a priori

Lastly, despite the fact that the work organization possibilities may be computed or described, the possibilities are regarded as emergent, consistent with the observations of Rochlin et al. (1987). First, the number of possibilities for a complex system is likely to be so large that it is not feasible for all of the possibilities to be considered meaningfully by analysts or designers. Certainly, this was found to be the case with the future maritime surveillance aircraft. Therefore, the possibilities for work organization can only be enacted meaningfully *in situ* by actors responding to local contingencies. Furthermore, although the work organization possibilities can be computed at some level, all of the details of these possibilities, including the local interactions between actors in the system, cannot be known or pre-specified. In fact, each fundamental possibility may have many new properties as it is enacted *in situ* by actors each time. Finally, the possibilities are regarded as emergent because it cannot be planned a priori which of the possibilities will be appropriate in unanticipated situations, as the details of these events cannot be known ahead of time. Even in situations that are regarded as familiar, there are likely to be many small variations in context that make prediction difficult. Therefore, typically only actors can enact sensibly particular possibilities for work organization from the fundamental set, in response to the local context, and thus finish the design.

### Design

Following the analytic effort to create a diagram of work organization possibilities for the actors in the system is the design phase. This section discusses how this diagram, or the set of work organization possibilities, can be utilized in design. First, the overarching design objectives are described. Subsequently, the design of particular elements is considered, specifically by illustrating how existing design approaches for individual elements, with complementary objectives, may be extended for the purposes of creating an integrated system design.

**Figure 11:**
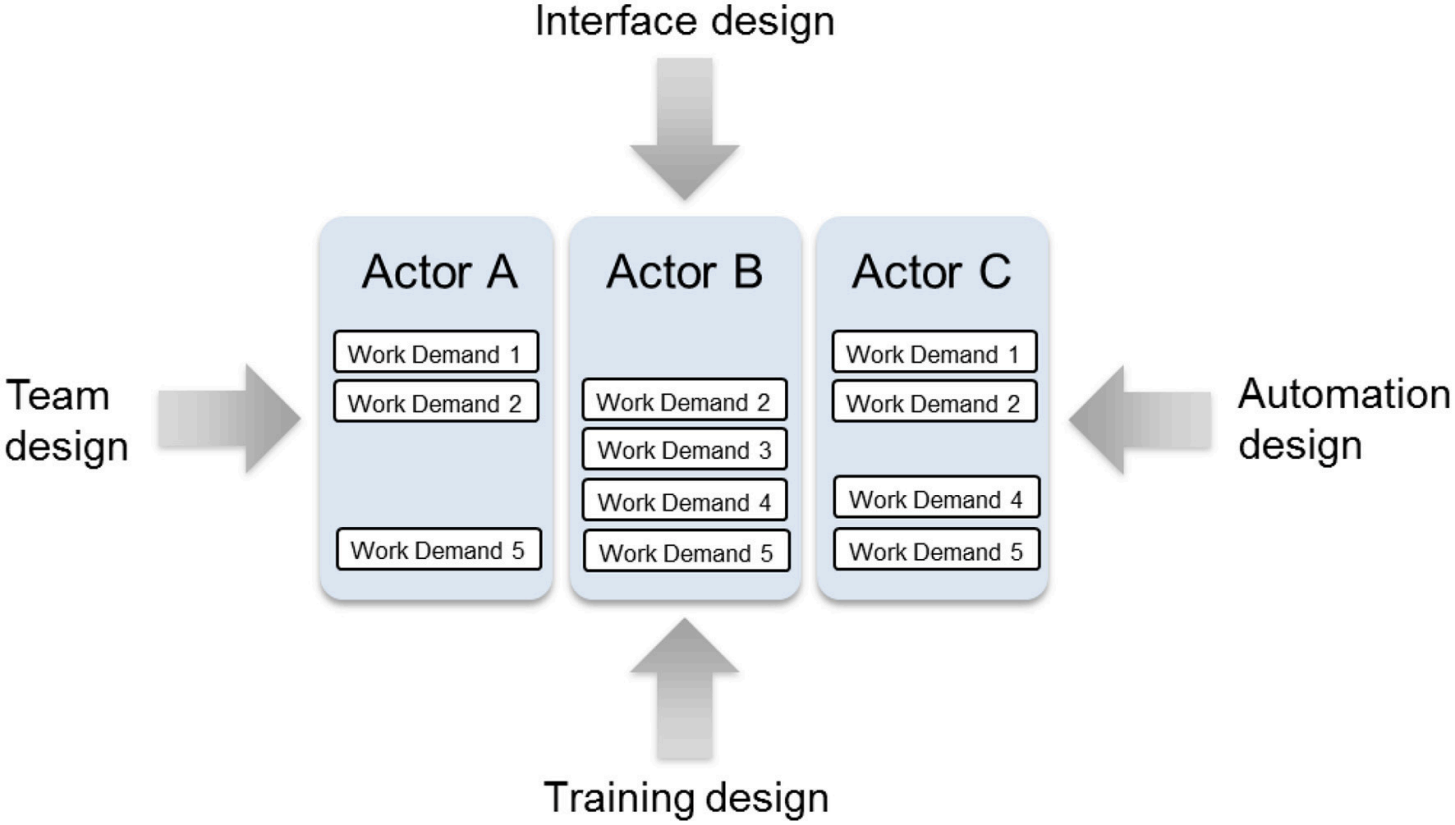
**The design of each system element must support the range of work organization possibilities**.

In the proposed approach, the aim of design—of each of the system elements—is to support the set of work organization possibilities, as identified in the WOP diagram. This idea is encapsulated in Figure 11. This figure conveys that the team and interface designs should be such that the range of possibilities for work organization can be adopted. Similarly, the automation and training designs should support this set of possibilities. In this way, the proposed approach anchors the design of these and other elements to the organizational constraints, so that multiple actors are supported in adapting their structure as well as their behavior in a coordinated manner, regardless of the situation.

Key to this principal objective is the idea that the design of each element should not artificially constrain the capacity of the system for adaptation. That is, the designs should not incorporate extraneous constraints, or constraints beyond those fundamental to the system, such that they limit unnecessarily the possibilities for work organization. For example, the roles of actors in a team should not be so construed that the team design eliminates reasonable alternatives for distributing the work demands across actors. Likewise, the information content of the displays made available to actors should not constrain the responsibilities each can adopt, by presenting information limited to a relatively narrow range of work demands, such that the set of possibilities for work organization in the system is constricted without reason.

Also central to this design perspective is the idea that the design of each element should seek to promote the capacity of the system for adaptation by alleviating any challenges or difficulties associated with realizing or executing the possibilities. As an example, if the suite of work demands for which an actor can be responsible requires considerable competencies, consideration should be given to how the learning demands can be managed through design, perhaps of the team and training program. It may be feasible, for instance, for the actor to serve as a deputy to a more senior position within the team, following some basic instruction, such that the full set of competencies for the job can be matured gradually through on-the-job training. Alternatively, if the combination of work demands for which an actor can be responsible entails substantial workload, emphasis could be placed on reducing the cognitive effort required for particular activities through the design of the display or automated decision aids. Finally, if the array of work demands for which an actor can be responsible involves significant coordination with other actors, consideration could be given to how the communication demands can be eased through the design of the workspace layout or collaboration technologies.

Within this overarching framework, complementary design approaches may be extended to develop the various elements. For example, ecological interface design (Rasmussen and Vicente, 1989; Vicente and Rasmussen, 1990, 1992; Burns and Hajdukiewicz, 2004; Bennett and Flach, 2011) can be extended to support the development of the interface by including the delineation of work organization possibilities. As this approach stands currently, a work domain analysis [and activity analysis, if one assumes the process Bennett and Flach (2011) describe] is conducted with the goal of identifying information requirements for displays for an actor, or actors, in the system. With the view of creating an integrated system design, however, a work domain analysis would be conducted also with the intention of demarcating the set of work organization possibilities. The work domain model would still be used to derive information requirements for displays, much like in the original approach, but with a key difference being that the information requirements would be based on the set of work demands for which each actor can be responsible, as established in the WOP diagram. These information requirements would be incorporated into the displays in a way that supports skill-based, rule-based, and knowledge-based behavior, as consistent with the original approach. The resulting interface, then, would provide workers with the information necessary for fulfilling the range of responsibilities they can adopt, not just those they are allocated or usually adopt, for instance in recurring classes of situation.

Notably, both Rasmussen et al. (1994) and Vicente (1999) recognize the importance of the work organization for design. This is reflected prominently in the fact that CWA, as described in these texts, includes a social organization and cooperation dimension. Moreover, Vicente, as a case in point, states explicitly that “the division and coordination of work determines what information content actors need to perform their duties” and that “making decisions about how work demands should be divided up has important implications for the identification of relevant information content” (p. 254). However, the existing approach for ecological interface design does not address how the implications of the work organization for the interface design can be derived systematically and, more specifically, in a formative manner. Therefore, while this approach can support adaptations in actors' behavior, in its current form it does not necessarily accommodate adaptations in their structure nor support the corresponding behavioral possibilities. Furthermore, it does not necessarily facilitate the integration of the interface with other elements, across multiple actors in the system, such that the range of possibilities for adaptation is supported in a coherent manner by the system design. The approach proposed here provides a means for addressing the organizational constraints in the design of the interface element.

Similarly, an existing approach utilizing CWA for team design (Naikar et al., 2003; Naikar, 2013) can be expanded to incorporate the delineation of work organization possibilities. While this approach does attempt to accommodate flexibility in the work structure through the team design, it is limited in its capacity to promote adaptation. Specifically, for a given system, work domain analysis and activity analysis are used to explore the feasibility of alternative team concepts, or alternative possibilities for work organization, in the context of plausible scenarios. On this basis, the strengths and limitations of the alternative concepts are identified, and requirements are generated for a new team design with the intent of capitalizing on the various possibilities. One limitation of this approach is that it relies on pre-conceived team concepts that are not necessarily constraint-based, such that it limits artificially the work organization possibilities that are considered. Moreover, the alternative team concepts are considered initially in the context of plausible situations, albeit both common and exceptional ones. Notably, this approach does involve generalizing beyond the particular situations examined, by translating the work demands in the scenarios into recurring work functions from the contextual activity template and by examining the impact of the alternative team concepts on the work domain constraints, which are relevant to a broad range of events including unforeseen ones. Nevertheless, a more parsimonious solution would be beneficial.

The approach proposed here provides a way of using CWA to generate the set of possibilities for work organization independently of the situation, within the constraints of the system, as captured in the WOP diagram. As a result, the requirements for the team design, such as the number, roles, and hierarchical levels of people in the team, can be defined in light of the suite of work demands actors can fulfill, regardless of the circumstances they find themselves in. Aside from accommodating greater possibilities for behavioral and structural adaptation, this team design can be integrated with other elements, across multiple actors, such that the possibilities are supported uniformly throughout the system.

An approach for using CWA for training design (Naikar and Sanderson, 1999; Naikar, 2013) also can be broadened to take into account the set of work organization possibilities. The current approach seeks to foster adaptation by promoting the design of training systems that offer the same possibilities for action that are afforded by the work environment or work domain. For example, a simulator with parallel means-ends structure to the work domain, or structural means-ends fidelity, will allow workers to exploit the same means-ends relations that are available in their actual work context. Hence, with the aid of a suitable training program, workers can become proficient in exploiting flexibly multiple system means, or resources, to fulfill multiple system ends, or purposes, such that they can respond in novel or adaptive ways to handle abnormal or unpredictable situations. Thus, in this approach, work domain analysis is central for defining the features or characteristics of training equipment or devices, such as high-fidelity simulators, whereas the remaining CWA dimensions provide a strong foundation for defining complementary training programs, although each dimension can inform either problem (see also Lintern and Naikar, 2000; Jenkins et al., 2011).

These ideas may be expanded for the purposes of creating an integrated system design. In developing training equipment or devices, consideration must be given to those physical and intentional features of the work domain that constrain or afford the work organization possibilities that are available to workers in their actual work context. In the case of high-fidelity simulators particularly, it may be desirable to recreate these properties so that workers have the same possibilities for work organization during training that are available to them otherwise. Similarly, training programs should give consideration to the full set of work demands that actors can assume responsibility for in their actual work context, as specified in the WOP diagram, so that workers are more suitably prepared for exploiting the range of possibilities for adaptation.

Finally, frameworks for automation design that are concerned principally with human-automation coordination (Dekker and Woods, 2002; Klein et al., 2004; Hollnagel and Woods, 2005; Woods and Hollnagel, 2006; Bradshaw et al., 2013) can be extended to take into account the set of work organization possibilities. Although these frameworks do not intrinsically involve the use of CWA, they are consistent with a constraint-based perspective in some respects. These frameworks recognize that the conventional preoccupation with the allocation of functions between humans and machines is limited and that the primary question of concern is not what level of autonomy or control is to be assigned to the human vs. the machine but rather, given the capabilities of the automation, how to support the interaction that would necessarily be required between humans and machines, if the capabilities of the automation are to be exploited.

This viewpoint aligns with the proposed approach for integrated system design. In relation to automation design specifically, the proposed approach recognizes that rather than focusing on pre-specifying a limited number of schemes for allocating work demands between humans and machines, which would inevitably be limited to anticipated events, it is necessary to identify the work demands that can be handled by the automation, alongside the human actors, irrespective of the situation. Subsequently, the interaction demands associated with the set of possibilities for work organization, encompassing both humans and machines, can be supported through design. Therefore, in the analysis phase, the automated agents can be treated as actors, as originally recognized by Rasmussen et al. (1994) and Vicente (1999), such that the set of work organization possibilities encompasses the potential distributions of work demands across human and machine actors. As per the earlier discussion, which possibility is adopted by humans, as only humans can take ownership of problems (Bradshaw et al., 2013), is dependent on the situation, such that sometimes the work structure might include both human and machine actors and sometimes not. Hence, the key implication for the design phase is the need to ensure that any one of the possibilities encapsulated in the WOP diagram can be implemented effectively, specifically by supporting the interaction demands associated with the range of work arrangements.

It is important to point out that the preceding discussion does not address all of the nuances in the implications of the integrated system design approach for the design of individual components. Nor does it address the full range of elements. This is beyond the scope of this paper. Rather, the intent has been to provide an illustration of how some existing, complementary design approaches for individual elements can be extended for the purposes of creating an integrated system design. Through these extensions the design of multiple elements can be anchored to, and thus coordinated around, the set of possibilities for work organization, such that the system design supports the structural and behavioral opportunities for adaptation systematically across multiple actors.

## Conclusion

To conclude, this paper has proposed an approach for integrated system design, based on extensions of CWA. This approach recognizes that to promote the capacity of sociotechnical systems for adaptation, the designs of the various elements must be integrated, such that workers are supported in adapting their structure as well as their behavior in a coherent manner. To this end, the approach proposes the set of possibilities for work organization in a system as the central mechanism for coordinating the design of multiple elements across multiple actors. Accordingly, the paper demonstrates how the set of work organization possibilities may be demarcated independently of the situation and how the resulting diagram of work organization possibilities may be utilized in design. Relative to existing analysis and design frameworks, this approach has the potential to enhance a system's capacity for adaptation by accommodating possibilities for structural adaptation across a variety of situations including unforeseen ones, supporting opportunities for behavioral adaptation associated with those structural possibilities, and facilitating the integration of multiple elements such that the system design supports the range of possibilities for adaptation, across multiple actors, in a systematic fashion.

As noted at the outset, the rationale for the proposed approach rests on the assumption that the principal design objective for sociotechnical systems should be that of facilitating successful adaptation, as these systems are open to changing conditions, including unanticipated events, which pose the most substantive threats to their viability. Moreover, supporting worker adaptation in everyday and novel situations is important not just for preserving system safety but also for promoting organizational productivity and workers' health. As ongoing adaptation to dynamic and unforeseen conditions is a highly exacting mode of operating, workers should be supported—deliberately and systematically—in adapting to the demands of the entire range of events through the system design. In particular, as consistent with considerable empirical evidence, the system design should support workers in adapting both their organization and behavior to the changing circumstances.

The proposed approach recognizes that current approaches for work analysis and design are limited in their capacity to support adaptation, for the most part, because they are focused on specifying optimal ways of performing work or describing existing ways of performing work under particular conditions, whether these are familiar, recurring, or anticipated. The CWA framework circumvents this limitation by focusing on the constraints on actors, rather than on the details of their work practices, as these constraints are relevant to both known and novel events. However, while this framework can support adaptation, as has been demonstrated empirically, the adaptations are constrained primarily to actors' behavior and do not necessarily extend to their work structure and the corresponding behavioral possibilities. A related problem is that CWA is limited in its ability to support integrated system design. Given that the design of various elements must be integrated across multiple actors in the system, understanding the possibilities for structural adaptation is necessary, and CWA, in its present form, does not provide a means by which this can be achieved comprehensively. The approach for integrated system design addresses these issues in the manner summarized above.

In closing, it is important to acknowledge that while the approach for integrated system design described in this paper has been demonstrated conceptually, its viability has not been fully established. Considerably more work is necessary to achieve this goal (Naikar and Elix, 2016b). A key objective of future research should be to validate the various ideas constituting this approach, either through experimental studies or case studies. Another important question to be addressed relates to the feasibility of implementing the complete approach, or aspects of it, in industrial contexts. By providing a comprehensive description of the approach for integrated system design, this paper enables these critical objectives to be pursued.

## Author contributions

NN: Substantial contributions to the conception or design of the work; or the acquisition, analysis, or interpretation of data for the work (specifically development of ideas, analysis, and writing); and drafting the work or revising it critically for important intellectual content; and final approval of the version to be published; and agreement to be accountable for all aspects of the work in ensuring that questions related to the accuracy or integrity of any part of the work are appropriately investigated and resolved. BE: Substantial contributions to the conception or design of the work; or the acquisition, analysis, or interpretation of data for the work (specifically analysis contributing to the development of ideas); and drafting the work or revising it critically for important intellectual content; and final approval of the version to be published; and agreement to be accountable for all aspects of the work in ensuring that questions related to the accuracy or integrity of any part of the work are appropriately investigated and resolved.

### Conflict of interest statement

The authors declare that the research was conducted in the absence of any commercial or financial relationships that could be construed as a potential conflict of interest.
